# Increasing participation in the 2019 UK general election amongst patients on a high intensity rehabilitation ward

**DOI:** 10.1192/bjo.2021.608

**Published:** 2021-06-18

**Authors:** Aaron Wood, Amrith Shetty

**Affiliations:** Bowmere Hospital, Peasley Cross Hospital

## Abstract

**Aims:**

To increase participation in the 2019 UK general election amongst inpatients on a high intensity rehabilitation ward, by supporting patients to both register to vote (RTV) and vote.

**Background:**

In 2000, the franchise was extended to those under section 2 or 3 as well as informal inpatients. Unfortunately, voting rates remain low: studies of the 2010 general election show voting rates amongst psychiatric inpatients to be 14%, compared to 65% for the general population. Engaging patients in the democratic process is not only just, it has been shown to be an effective avenue for rehabilitation through increasing social capital. The 2019 UK general election represents a singular opportunity for biopsychosocial rehabilitation.

**Method:**

In the three weeks up until 26/11/19 – the deadline to RTV – visual displays and verbal information were used to notify patients of:

- The election
- Their eligibility
- The need to RTV before casting a ballot
- The registration deadline
- Voting methods (in person, by post, by proxy)

We gathered patients’ intention to RTV and offered impartial, personalised support to register online or by paper, and to apply for a postal or proxy ballot if wished. Patients with no fixed abode were supported to use the ward as their declared place of residence.

**Result:**

Of the 17 patients on the ward there were:

Four informal patients

11 patients under section 3

One patient each under a section 37 and a section 37/41, both ineligible to vote

Of the 15 eligible patients, one (6.7%) had already registered, six patients (40%) wanted to register and eight (53.3%) stated they did not want to register. Those wanting to register were supported according to individual patient preference. Of the registered seven, five (33.3%) reported voting, one (6.7%) reported not having voted and one (6.7%) declined to say. Two (13.3%) voted in person and five (33.3%) voted by postal ballot.

**Conclusion:**

Our intervention corresponded with an increase in number of patients registering – from one patient (6.7%) to seven (46.7%), with 5-6 (33.3-40%) casting their ballot. While the causal relationship should not be overstated, the uptake of assistance supports the intervention's efficacy.

Good rehabilitation increases a person's social capital, empowering them to actively participate in societal life. Registering to vote is a tacit assertion of this principle. Our study shows that brief interventions that are easily incorporated into everyday care are a simple, effective and ultimately necessary tool in holistic mental health rehabilitation.

